# A Meta-Analysis of Changes in Brain Activity in Clinical Depression

**DOI:** 10.3389/fnhum.2014.01045

**Published:** 2015-01-14

**Authors:** Susan M. Palmer, Sheila G. Crewther, Leeanne M. Carey, The START Project Team

**Affiliations:** ^1^Neurorehabilitation and Recovery, Stroke Division, The Florey Institute of Neuroscience and Mental Health, Melbourne Brain Centre, Heidelberg, VIC, Australia; ^2^School of Psychological Science, La Trobe University, Bundoora, VIC, Australia; ^3^Department of Occupational Therapy, School of Allied Health, La Trobe University, Bundoora, VIC, Australia

**Keywords:** depression, meta-analysis, fMRI, task activation, resting state, cognition, emotion, brain networks

## Abstract

Insights into neurobiological mechanisms of depression are increasingly being sought via brain imaging studies. Our aim was to quantitatively summarize *overlap and divergence* in regions of altered brain activation associated with depression under emotionally valenced compared to cognitively demanding task conditions, and with reference to intrinsic functional connectivity. We hypothesized differences reflective of task demands. A co-ordinate-based meta-analysis technique, activation likelihood estimation, was used to analyze relevant imaging literature. These studies compared brain activity in depressed adults relative to healthy controls during three conditions: (i) emotionally valenced (cognitively easy) tasks (*n* = 29); (ii) cognitively demanding tasks (*n* = 15); and (iii) resting conditions (*n* = 21). The meta-analyses identified five, eight, and seven significant clusters of altered brain activity under emotion, cognition, and resting conditions, respectively, in depressed individuals compared to healthy controls. Regions of overlap and divergence between pairs of the three separate meta-analyses were quantified. There were no significant regions of overlap between emotion and cognition meta-analyses, but several divergent clusters were found. Cognitively demanding conditions were associated with greater activation of right medial frontal and insula regions while bilateral amygdala was more significantly altered during emotion (cognitively undemanding) conditions; consistent with task demands. Overlap was present in left amygdala and right subcallosal cingulate between emotion and resting meta-analyses, with no significant divergence. Our meta-analyses highlight alteration of common brain regions, during cognitively undemanding emotional tasks and resting conditions but divergence of regions between emotional and cognitively demanding tasks. Regions altered reflect current biological and system-level models of depression and highlight the relationship with task condition and difficulty.

## Introduction

Depression is a major burden to society currently affecting an estimated 350 million people globally (World Health Organization, 2012). The lifetime prevalence varies widely, from 3% in Japan to 17% in the USA, with most countries in the range of 8–12% (Andrade et al., 2003). A recent survey across 17 countries found that on average 1 in 20 people report having an episode of depression in the previous year (World Health Organization, 2012). The definition for clinically depressed patients used in this meta-analysis included a current diagnosis of depression with a criterion of at least five of the following symptoms most days for at least 2 weeks: depressed or irritable mood, decreased interest or pleasure in daily activities, significant weight change, changes to sleep patterns, changes in activity patterns, fatigue or loss of energy, feelings of guilt/worthlessness, diminished concentration, and thoughts of suicide (American Psychiatric Association, 2013).

In recent times, a rapidly growing number of brain imaging studies, including positron emission tomography (PET) and functional magnetic resonance imaging (fMRI), have reported differences in induced task activations between patients with depressive symptoms and control subjects when performing specified tasks [e.g., Dichter et al. (2009), Hsu et al. (2010), Carballedo et al. (2011), van Eijndhoven et al. (2011), Sexton et al. (2012)]. There are also reported differences when measuring functional connectivity, i.e., coherence of signal based on temporal similarities, between brain regions during a resting-state condition [see Stuhrmann et al. (2011), Wang et al. (2012), for recent review].

In 2008, a comprehensive meta-analysis by Fitzgerald et al. (2008a) quantitatively summarized brain regions with increased or decreased brain activation in depressed patients compared to healthy controls using the activation likelihood estimation (ALE) method. A number of “hypoactive” regions were identified in depressed patients, including frontal and temporal cortex, during emotion tasks. In addition, a relative increase in activity was identified in subcortical and limbic regions. An increase in activity was also reported in the insula and cerebellum with treatment. Unfortunately, at that time, there were few imaging publications involving cognitive tasks and tasks were not considered sufficiently homogeneous for formal analysis. Interestingly, a paper from the same group (Fitzgerald et al., 2008b) involving direct comparison of depressed patients and controls performing the Tower of London and an *n*-back cognitive task, reported greater activation in right prefrontal cortical regions. This leads the authors to suggest that “patients with depression may recruit greater brain regions to achieve similar or even poorer task performance than control subjects.” Today, there are 15 additional imaging publications involving cognitive tasks (Table 1). Hence, we wish to reanalyze the studies in the Fitzgerald meta-analysis and the 15 additional imaging publications to further explore the relationship between activation under cognition compared to emotion task conditions.

**Table 1:** **Table of included studies**.

Article	No of participants	Imaging mode	Task	Patient depression score mean (SD)	Mean (SD) patient age	Medicated
**EMOTION**						
Bremner et al. (2007)	18D:9HC	PET – [^15^O] H_2_O	Emotional words (sad/depressive) – recall of words	16 (7)^a^	40 (13)	No
Canli (2004)	15D:15HC	fMRI	Emotional words (sad/socially or physically threatening/happy)	23.9 (7.51)^c^	35.1	7/15 on medication
Carballedo et al. (2011)	15D:15HC	fMRI	Emotional face (sad/angry) – matching task	22.87 (4.35)^a^	39.87 (8.57)	No
Dichter et al. (2009)	14D:15HC	fMRI	Emotional (sad) and neutral pictures	26.9 (4.9)^a^	34.8 (14.3)	No
Elliot (2002)	10D:11HC	fMRI	Emotional words (happy/sad) with neutral distraction	23.1 (3.9)^a^	42.2 (8.3)	Yes
Epstein et al. (2006)	10D:12HC	fMRI	Emotional words (positive/negative/neutral) – review	ns	35.6	No
Fu et al. (2004)	19D: 19HC	fMRI	Emotional faces (sad) – view, asked to nominate gender	21.1 (2.3)^a^	43.2 (8.8)	No
Fu (2007)	19D:19HC	fMRI	Emotional faces (happy) – view, asked to nominate gender	21.1 (2.3)^a^	43.2 (8.8)	No
Gotlib et al. (2005)	18D:18HC	fMRI	Emotional faces (sad/happy) – view, asked to nominate gender	24.6 (8.3)^c^	35.2	9/18 on medication
Grimm et al. (2008)	19D:29HC	fMRI	Emotional pictures – judge positivity/negativity	33.12^a^ (7.13)	40.00 (9.89)	Yes
Grimm et al. (2009)	25D:25HC	fMRI	Emotional pictures – judge self-relatedness of positive and negative pictures	26.8^a^	ns	Yes
Hsu et al. (2010)	23D:20HC	fMRI	Emotional words (positive/negative/neutral) – read and respond if word was understood	19.41 (2.63)^a^	41.26 (11.67)	No
Keedwell et al. (2005)	12D:11HC	fMRI	Emotional events (happy/sad/neutral) in subject’s life – reminded of	33.5 (11.2)^c^	43 (9.8)	11/12 on medication
Kumari et al. (2003)	6D:6HC	fMRI	Emotionally captioned pictures (positive/negative) – viewed	19.33 (1.03)^a^	47 (3.59)	ns But all patients cited as being treatment resistant
Lawrence et al. (2004)	9D:11HC	fMRI	Emotional faces (sad/fearful/happy) – view, asked to nominate gender	31.8 (11.8)^c^	41 (11)	Yes
Mingtian et al. (2012)	27D:25HC	fMRI	Emotional faces (fearful/angry) – matching task	25.11 (5.42)^d^	20.37 (1.86)	No
Mitterschiffthaler et al. (2003)	7D:7HC	fMRI	Pictures – assess positivity of positive and neutral pictures	33.6 (2.5)^c^	46.3 (8.1)	Yes
Mitterschiffthaler et al. (2008)	17D:17HC	fMRI	Emotional (sad/neutral) STROOP test	20.88 (1.83)^a^	39.3 (9.4)	No
Ritchey et al. (2011)	22D:14HC	fMRI	Pictures – rate “pleasantness” of pictures (positive/negative/neutral)	26.7 (6.7)^a^	36.1 (10.1)	Yes
Siegle et al. (2002)	7D:10HC	fMRI	Words – assess the emotion of positive, negative, and neutral words	21.6 (9.9)^c^	34.3 (8.8)	No
Siegle et al. (2007)	20D:21HC	fMRI	Emotional words (positive/negative/neutral) – assess personal relevance of	27.3^c^	38.8	No
Surguladze et al. (2005)	16D:14HC	fMRI	Emotional faces (happy/sad/neutral) – view, asked to nominate gender	31.1 (10.8)^c^	42.3 (8.4)	ns
Surguladze et al. (2010)	9D:9HC	fMRI	Emotional faces (fear/disgust) – view, asked to nominate gender	31.8 (11.8)^c^	42.8 (7.2)	Yes
Townsend et al. (2010)	15D:15HC	fMRI	Emotional faces (sad/fearful) – matching task or emotion identification	20.1 (4.9)^a^	45.6 (11.2)	No
Tremblay et al. (2005)	12D:12HC	fMRI	Pictures – rate “pleasantness” of positive, negative, and neutral pictures	27.75 (3.05)^a^	34.8 (13.96)	No
Wang et al. (2008a)	19D:20HC	fMRI	Emotional faces (sad/neutral) – view, asked to detect circle associated with sad pictures	19.9 (5.3)^a^	39.3 (9.0)	11/19 on medication
Wang et al. (2008b)	12D:20HC	fMRI	Emotional faces (sad/neutral) – view, asked to detect circle	23.7 (5.7)^b^	69.1 (6)	9/12 on medication
Yoshimura et al. (2010)	13D:13HC	fMRI	Emotional words – self-referential judgment of positive and negative personality words	26.5 (6.9)^c^	37.6 (6.2)	Yes
Zhong et al. (2011)	29D:31HC	fMRI	Emotional faces (fearful/angry) – matching task	34.86 (5.41)^d^	20.45 (1.82)	No
**COGNITION**						
Audenaert et al. (2002)	10D:10HC	SPECT – ^99m^Tc ECD	Letter fluency test and category fluency test	27 (3.8)^a^	19–49 year	Yes
Bremner et al. (2004)	18D:13HC	PET – ^99m^Tc ECD	Verbal memory encoding task remembering words	33 (9)^a^	38 (2)	Yes
de Asis et al. (2001)	6D:5HC	PET – ^99m^Tc ECD	Paced word generation	>30^a^	70.7	4/6 on medication
van Eijndhoven et al. (2011)	20D:20HC	fMRI	Episodic memory task	21.08 (4.03)^a^	34.1 (11.6)	Yes
Elliott et al. (1997)	6D:6HC	PET – ^99m^Tc ECD	Tower of London task	23.8^a^	34.7	5/6 on medication
Fitzgerald et al. (2008b)	13D:13HC	fMRI	Tower of London, *n*-back letter memory task (where *n* = 2)	32.7 (11.9)^b^	38.4 (8.1)	11/13 on medication
Harvey et al. (2005)	10D:10HC	fMRI	*N*-back working memory (where *n* = 0, 1, 2, 3)	26.7 (4.6)^b^	33.8 (8.4)	5/6 on medication
Holmes et al. (2005)	10D:9HC	fMRI	AX expectancy performance task. Subjects asked to respond to letters in sequence, e.g., AX	ns	32 (9.87)	ns
Hugdahl et al. (2004)	12D:12HC	fMRI	Mental arithmetic	22.6 (3.5)^a^	32.8 (8)	Yes
Matsuo et al. (2007)	15D:15HC	fMRI	*N*-back working memory (where *n* = 0, 1, 2)	20.3 (5.3)^a^	34.3 (11.5)	No
Okada et al. (2003)	10D:10HC	fMRI	Verbal fluency (making words from Japanese phonetic characters)	19^a^	46.6 (7.9)	Yes
Remijnse et al. (2009)	20D:27HC	fMRI	Reversal learning (learning from positive/negative feedback)	19.1 (4.1)^a^	35	Yes
Taylor Tavares et al. (2008)	13D:15HC	fMRI	Reversal learning (learning from positive/negative feedback)	13 (1.6)^a^	38.3 (2.3)	ns
Werner et al. (2009)	11D:11HC	fMRI	Associative learning paradigm	20.27 (8.74)^c^	37.18 (10.35)	8/11 on medication
Young et al. (2012)	12D:14HC	fMRI	Recall of memory triggered by positive, negative, and neutral words	21^a^ (8.3)	34 (11)	No
**RESTING**						
Bench et al. (1992)	23D:33HC	PET – C_15_O_2_	Resting	25 (4.1)^a^	56.8 (12.8)	19/33 on psychotrophic medication
Brody et al. (2001)	14D:15HC	PET – ^18^F	Resting	19.4 (5.4)^a^	38.9 (11.4)	No
Drevets et al. (1992)	13D:13HC	PET – [^15^O] H_2_O	Resting	27.3 (4.6)^a^	36.2 (8.9)	No
Duhameau et al. (2010)	6D:6HC	ASL	Resting	22.5 (4.97)^a^	52.5 (8.67)	Yes
Greicius et al. (2007)	28D:20HC	fMRI	Resting	25.4 (4)^a^	38.5	20/28 on medication
Ito et al. (1996)	11D:9HC	SPECT – ^99m^Tc HMPAO	Resting	10.6 (7.9^a^	66.6 (7.1)	Yes
Kennedy et al. (2001)	13D:24HC	PET – ^18^F	Resting	>18^a^	36 (10)	No
Kimbrell et al. (2002)	37D:37HC	PET – ^18^F	Resting	16.9^a^ (8.3)	43.4 (13)	Yes
Liu et al. (2010)	15D:15HC	fMRI	Resting	32.6^a^ (6.5)	29.13 (13.55)	No
Mayberg et al. (2005)	6D:6HC	PET – [^15^O] H_2_O	Resting	25.8^a^ (2.8)	46 (8)	Yes
MacHale et al. (2000)	12D:15HC	fMRI	Resting	24.0^a^	44.3 (12.5)	10/12 on medication
Monkul et al. (2012)	20D:21HC	PET – [^15^O] H_2_O	Resting	>18^a^	37.2 (13.6)	No
Oda et al. (2003)	23D:13HC	SPECT – ^99m^Tc ECD	Resting	21.2 (13.7)^a^	54.5 (7)	Yes
Peng et al. (2011)	16D:16HC	fMRI	Resting	>20^a^	34.1 (9.2)	No
Périco et al. (2005)	15D:15HC	SPECT – ^99m^Tc ECD	Resting	26.9 (6.5)^a^	34.5 (10.2)	Yes
Saxena et al. (2001)	27D:17HC	PET – ^18^F	Resting	20.8 (5)^a^	38.2 (11.1)	Yes
Skaf et al. (2002)	12D:12HC	SPECT – ^99m^Tc ECD	Resting	26.91 (6.52)^a^	41.44 (10.67)	Yes
Veer et al. (2010)	19D:19HC	fMRI	Resting	14.21 (9.62)^b^	36.2 (9.7)	No
Videbech et al. (2001)	42D:47HC	PET – [^15^O] H_2_O	Resting	>17^a^	41.9 (12.7)	No
Wu et al. (2011)	22D:26HC	fMRI	Resting	23.2 (4.8)^a^	35 (13)	No
Yao et al. (2009)	22D:26 HC	fMRI	Resting	<35^a^	38.2 (10.2)	Yes

^a^HAMD Scale 0–56;

^b^MADRS Scale 0–60;

^c^BDI Scale 0–63;

*^d^CES-D Scale 0–60*.

*D, depressed patients; HC, healthy controls; IAP, international affective picture system; MDD, major depressive disorder; ns, not specified*.

*Mean patient age is the mean age of the depressed patients. SD is included in brackets if quoted. [^15^O] H_2_O, radioactively labeled water; ^99m^Tc ECD, l,l-ethyl cysteinate dimmer labeled with technetium-99m; C_15_O_2_, carbon dioxide labeled with ^15^C; ^99m^Tc HMPAO, hexamethylpropyleneamineoxime labeled with technetium-99m*.

In 2012, Diener et al. (2012) performed an ALE meta-analysis that combined data from tasks defined in the original study as either emotional or cognitive. They found predominantly hypoactive clusters in anterior insula and anterior cingulate cortex, with activation alterations of frontal regions as well as thalamus and striatum. In comparison, meta-analyses of resting-state activity in depressed patients compared to controls found altered activity in bilateral thalamus (Hamilton et al., 2012) as well as striatal and cortical areas (Kühn and Gallinat, 2013). Graham et al. (2013) used ALE and Gaussian-process regression analyses of emotional, cognitive, and resting conditions to compare current neural models of depression outlined below and showed broad support for the limbic-cortical and corticostriatal models of depression.

Over the last decade, at least three non-mutually exclusive neurobiological models of major depressive disorder have been described: limbic-cortical, corticostriatal, and the default mode network. Mayberg (2003) outlined a limbic-cortical model that suggests depression is linked to over-activity in limbic areas traditionally associated with emotional processing and inadequate inhibition by prefrontal areas (Mayberg et al., 1999). This well established model is understood to include lateral and medial prefrontal cortex, orbitofrontal cortex, anterior cingulate cortex, hippocampus, thalamus, and amygdala. The corticostriatal model emphasizes the role of subcortical structures in information processing, with parallel overlapping cortico-striato-pallaidal-thalamic loops, and striatal dysfunction associated with symptoms such as psychomotor retardation (Bora et al., 2012). Clinical and research literature also suggest down regulation of the attention-affected blood oxygenation level dependent (BOLD) response and increased activation when comparing goal-directed task activated areas associated with emotion and cognition conditions in clinically depressed individuals compared to controls (Drevets, 2000; Halari et al., 2009; Pizzagalli, 2011; Chiong et al., 2013; Li et al., 2014). An increased dominance of the default mode network (Raichle et al., 2001) has more recently been linked [Sheline et al. (2009), Hamilton et al. (2011), for review Berman and Jonides (2011), Holtzheimer and Mayberg (2011)] to symptoms of depression where individuals are reported to show increased self-reflective rumination, as opposed to adaptive reflective rumination or task-positive mode. In this context, we were interested in exploring the brain regions that are statistically *altered*, i.e., show increased or reduced BOLD activation (in context of emotion and cognition studies) or increased or reduced brain activity under resting conditions, in the limbic-cortical, corticostriatal, and the default networks. Our approach was to investigate this through the separate analysis of emotion, cognition, and resting imaging studies, and to identify commonalities and divergence of altered regions across study conditions. While it is recognized that neural substrates underlying different imaging methods, such as BOLD activation and correlation of BOLD fluctuations, may differ, prior meta-analyses support the investigation of neurobiological models based on a broad range of paradigms, including resting-state fMRI and PET (Fitzgerald et al., 2008a; Graham et al., 2013). Moreover, common patterns of distributed brain regions have been characterized despite the complexity and diversity of the imaging methods used (Fitzgerald et al., 2008a).

While it is likely that processing of emotion and cognitive tasks may involve common networks in both depressed patients and controls (Pessoa, 2014), behavioral criteria of depression would suggest that clinical patients need to apply greater conscious effort to successfully complete the same demanding tasks during a depressive episode than when healthy (Berman et al., 2010). Thus, it is reasonable to further hypothesize that tasks classified as cognitively demanding may induce increased activation in appropriate thalamic and frontal brain areas if similar performance level is to be achieved (Haier et al., 1988; Pessoa, 2014). Indeed, level of activation, i.e., hypoactivity or hyperactivity is reported to vary with clinical diagnosis and task difficulty (Carballedo et al., 2011; Diener et al., 2012).

Historically, classification of neuroimaging tasks used in the depression literature have included emotion and more complex cognitive tasks. “Emotion tasks” have been defined as those that required the subject to respond either explicitly or implicitly to emotional stimuli presented visually or verbally (see Table 1, for examples) with low cognitive demands. More difficult “cognitive tasks” included those requiring maintenance and/or manipulation of information in either short-term, long-term or working memory. We have selected only studies that compared patients assessed to be currently depressed by structured clinical interview for DSM IV disorders (First et al., 1995), with control subjects. Patients under remission were not included. DSMIV was used as the criterion as all the studies reviewed were published before the May 2013 release date of DSMV.

In summary, we aimed to review the current literature as a means of updating and further characterizing the effect of clinical depression on functional neural network activity during tasks classified as simple and more emotionally valenced or relatively more difficult cognitive based (Pessoa, 2014), and during resting conditions. Further, we now have the benefit of added features of an upgraded meta-analysis software with improved meta-analysis technique and functionality whereby the results of two meta-analyses can be compared (contrast analysis) (Eickhoff et al., 2011). Using this software, an estimation of the statistically significant areas of cluster overlap, indicative of commonality of activation or correlation differences between clinically depressed patients and healthy controls, can be quantified under several task conditions. Areas of divergence can also be estimated, indicative of differences in areas of activation by the two groups under different tasks or perhaps tasks that vary in difficulty. In particular, we aimed to explore and quantify regions of overlap and divergence in order to advance our understanding of the relative influence of depression on brain regions under these different conditions. Identification of common and divergent brain regions may better inform current neurobiological models of depression and the mechanisms underlying. Further, it may add to the growing support for common brain regions under emotionally valenced and cognitive conditions (Pessoa, 2008, 2014), in the context of depression. In relation to the direction of increased or decreased differences (investigated via our exploratory meta-analysis), we expected differences in depressed individuals on the basis of *capacity limited* performance (Pessoa, 2014) suggesting that depressed individuals need to work harder to achieve the same results as normal healthy controls. Finally, based on the quantitative synthesis of brain regions altered in persons suffering from depression under emotion and cognition conditions and at rest, we sought to create brain maps of the regions of interest that may be employed in subsequent imaging analysis of changes in brain networks in populations with depression, such as following stroke.

## Materials and Methods

### Data used for meta-analysis

**Figure 1:**
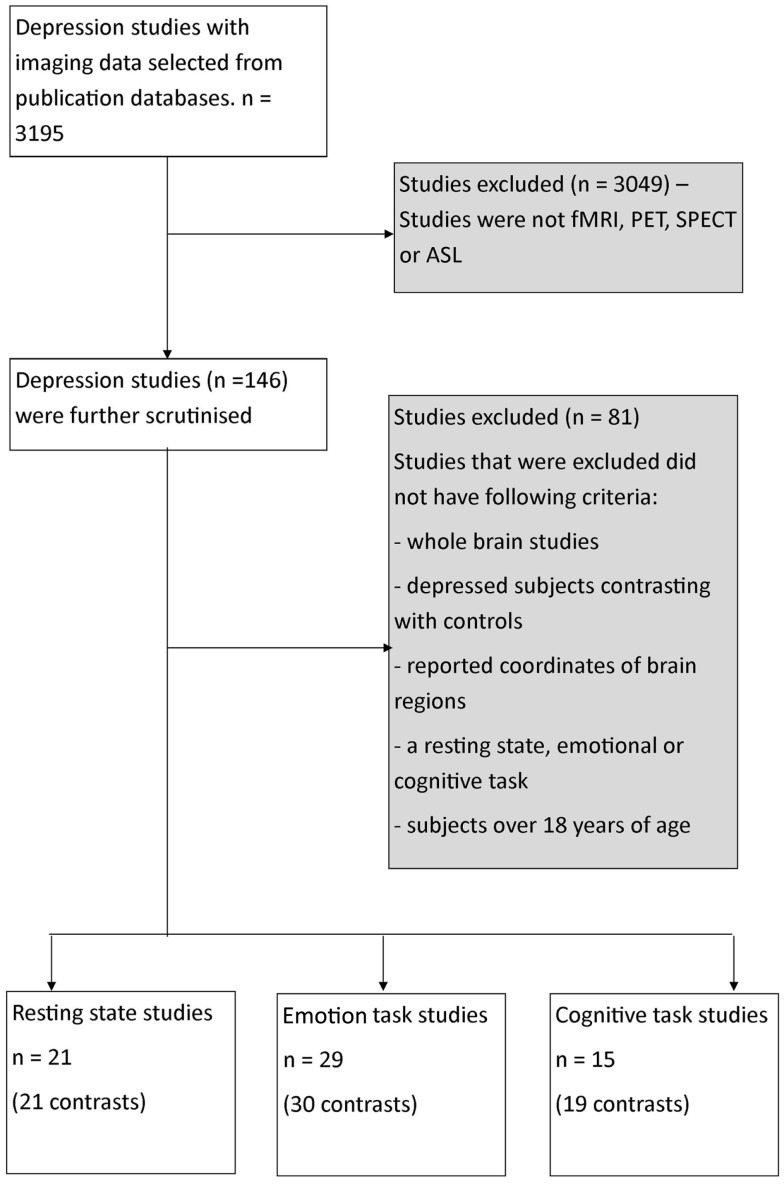
**Flowchart of process of selection of publications for meta-analysis**. fMRI, functional magnetic resonance imaging; PET, positron emission tomography imaging, SPECT, single-photon emission computed tomography imaging; ASL, arterial spin labeling imaging.

Our search included literature up until July 2013 from relevant databases including Medline, Web of Sciences, and PsycINFO using the following search term combinations – “fMRI,” “PET,” “resting-state MRI,” “depression,” as well as including articles obtained from the reference list of the selected articles that were reviewed. A total of 3579 articles were identified from the search (Figure 1).

The articles were then reviewed on the basis of strict inclusion and exclusion criteria. Only studies that involved participants with clinical depression (based on a range of assessment scales, see Results for assessment scales used), were compared with normal controls, involved whole-brain analysis, and reported activation or time series correlation co-ordinates in standard space were included. Studies that included a treatment or participants <18 years of age were excluded. Furthermore, all studies were required to utilize imaging techniques of similar temporal sampling including either PET, single-photon emission computed tomography (SPECT), arterial spin labeling (ASL) or functional MRI (fMRI) technology. They were also required to involve an emotionally valenced task (viewing items with emotional content with or without response) or a cognitive task (performing a mental processing task) or resting condition (measuring correlated brain activity or connectivity under non-specified rest condition). Only resting studies that used whole-brain data driven analysis approach such as independent component analysis (ICA) were included. Studies with seed-based approach to analysis were not included. In each study, brain co-ordinates were included where a *difference* (either increase or decrease in correlated brain activity) between the depressed patients and control subjects was reported. These brain co-ordinates were included as absolute difference co-ordinates in the *primary* meta-analyses. From a statistical viewpoint, the ALE uses a general linear model without consideration of sign of difference in activation or correlation. Co-ordinates were then further divided into whether the difference reported was an increase or a decrease in activity in depressed patients compared to healthy controls. This allowed us to conduct separate *directional* meta-analyses for emotion and cognition, or a relative increase or decrease in correlated activity for depressed compared to healthy control subjects for the resting studies.

Based on these criteria, a total of 65 studies were determined to be suitable for inclusion (Figure 1; Table 1). This amounted to a total of 29 emotional processing, 15 cognitive processing, and 21 resting condition publications for review. Studies were categorized into the respective groups based on the original author’s descriptive categorization and confirmed by consensus across two of the current authors. In addition, the type of task is briefly categorized in Table 1 of the results. Within the emotional processing publications, 17 studies were available to study areas where activation was increased and 20 studies available to study areas where activation was decreased. Furthermore, within the cognitive publications, 11 studies were available to study areas where activation was increased and 9 studies available to study areas where activation was decreased. Finally, within the resting publications, 13 studies were available to study areas where correlated activity was increased and 5 studies available to study areas where correlated activity was decreased. Separation of studies according to increased/decreased activation/correlation resulted in a reduced number of studies available for each meta-analysis, which would in turn affect the statistical power of the meta-analysis and have implications when performing contrast analyses across meta-analyses. Our purpose in pooling co-ordinates of *difference* activity for the *primary* analyses was to robustly determine spatial location of brain areas involved, irrespective of increased or decreased activity, so that these could be considered as regions of interest in future studies investigating regions disrupted by brain injury such as stroke. This approach also allowed us to conduct contrast analyses to determine regions of overlap and divergence. We pooled co-ordinates from areas of increased/decreased activity (as absolute difference locations) and thus performed a meta-analysis of brain areas that were altered in depressed subjects relative to controls for each emotional processing, cognitive processing, and resting meta-analysis. A *post hoc* exploratory analysis was conducted involving separate ALE analyses on the studies divided into increased or decreased activity relative to controls to provide an indication of the direction of change, while recognizing that the number of studies included in these meta-analyses was relatively low.

### Quantitative meta-analysis using ALE

The meta-analysis was performed using ALE on the co-ordinates of maximum activation/correlation of the brain regions reported by the selected study. Analysis was performed using GingerALE (version 2.1) software (downloaded from http://brainmap.org/ale) in Montreal Neurological Institute (MNI) space (Eickhoff et al., 2009, 2012). Co-ordinates reported in Talairach space were converted to MNI space using the icbm2tal transform (Lancaster et al., 2007; Laird et al., 2010).

To minimize within experiment and within group effects we utilized the modified algorithm described in Turkeltaub et al. (2012), which limits the effect of a single experiment. Thus, we were able to include multiple task studies (listed in Table 1) from within the one study. The calculated ALE map was thresholded at *p* < 0.05 [corrected for multiple comparisons using the false discovery rate (FDR)]. GingerALE provides a recommended cluster size, such that the cluster will not be made up entirely of false positives. This is calculated on the FDR and provides a means to threshold the clusters by size. Accordingly, the clusters were thresholded at 672 mm^3^ for the emotion meta-analysis, 368 mm^3^ for the cognition meta-analysis, and 376 mm^3^ for the resting meta-analysis. Different cluster sizes across studies are influenced by the number of studies, maximum peak, and statistical threshold. We were interested in identifying brain areas that were altered by depression, hence co-ordinates were selected for brain areas showing a difference in depressed patients compared to control subjects (either increased or decreased), implying a difference in brain processes in the depressed patients. Our directional meta-analyses of increased or decreased activation in depressed patients compared to healthy controls were also conducted at the recommended threshold corrected for multiple comparisons.

### Subtraction analysis

We also performed a subtraction contrast analysis for each of the three meta-analyses, combining two meta-analyses at a time (Eickhoff et al., 2011). In this approach, the two selected ALE analyses are statistically compared and the differences matched with a null-distribution. The null-distribution is generated by pooling all the experiments within the two ALE analyses and then randomly assigning the data to either of the comparison groups and reviewing these groups for 10,000 permutations. This generated *p* values at the voxel level, indicating significant difference or overlap. These were thresholded at *p* < 0.05. A cluster minimum of 10 mm^3^ was also applied to avoid reporting of incidental overlap or divergence.

Finally, we explored the potential for overlap across all three meta-analyses through an inspection and tabulation of outputs from each of the meta-analyses in order to describe common patterns. We conducted this descriptive analysis at the recommended cluster threshold nominated as well as at the more lenient and exploratory cluster size threshold of 100 mm^3^.

## Results

Following a review of the literature using the criteria listed in the methods, we included the papers listed in Table 1. As described in the Section “Materials and Methods,” these studies were grouped into emotion tasks, cognition tasks, or resting condition studies. The type of task (emotion, cognition, or resting) undertaken is listed in Table 1. The emotion related tasks primarily involved viewing, recognition, or matching of emotional faces (*n* = 12); assessing or matching emotional words (*n* = 7); assessing positivity/negativity or emotion of pictures (*n* = 6) or emotional STROOP (*n* = 1). The cognition tasks involved verbal and working memory tasks (*n* = 5), reversal/associative learning (*n* = 3), word fluency/generation (*n* = 3), Tower of London (*n* = 2), mental arithmetic (*n* = 1), or performance expectancy (*n* = 1). Thus, the emotion related tasks are cognitively easy while the cognitive tasks are much more demanding. Age of the depressed subjects was typically quoted as a mean age in each of the contributing studies. The overall mean age across the groups did not vary greatly, as the mean of the mean ages of the emotion and cognition and resting studies were 39.5, 38.4, and 41.8 years, respectively. Assessment for the inclusion of the subjects as depressed varied between a number of clinical tests including Hamilton rating scale for depression (HAMD) (Hamilton, 1960), Montgomery–Asberg depression rating scale (MADRS) (Montgomery and Asberg, 1979), Beck depression inventory (BDI) (Beck et al., 1961), and Center for Epidemiologic Studies Depression Scale (CES-D) (Radloff, 1977).

**Figure 2:**
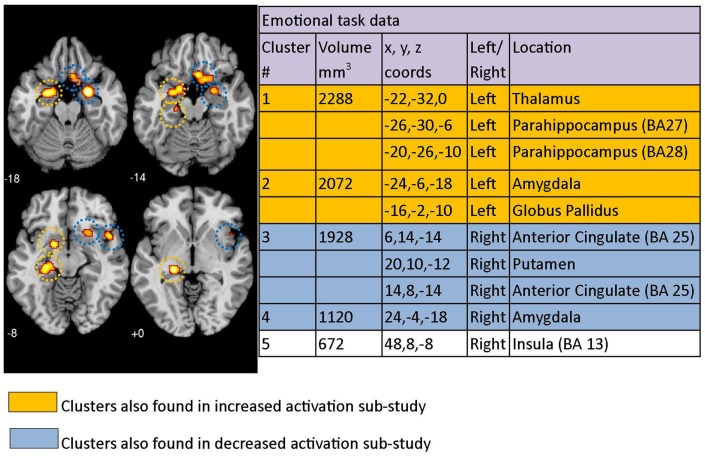
**Regions where depressed patients were altered in activity patterns compared to controls when performing an emotional processing task**. Clusters listed in the table are taken from the primary meta-analysis comprising all altered co-ordinates. Cluster regions highlighted in orange signify brain areas where depressed patient activation was increased compared to controls. Cluster regions highlighted in blue signify brain areas where patient activation was decreased compared to controls. Co-ordinates (*x*, *y*, *z*) are reported in MNI space. The number under each brain slice displayed is the MNI “*z*” co-ordinate.

**Figure 3:**
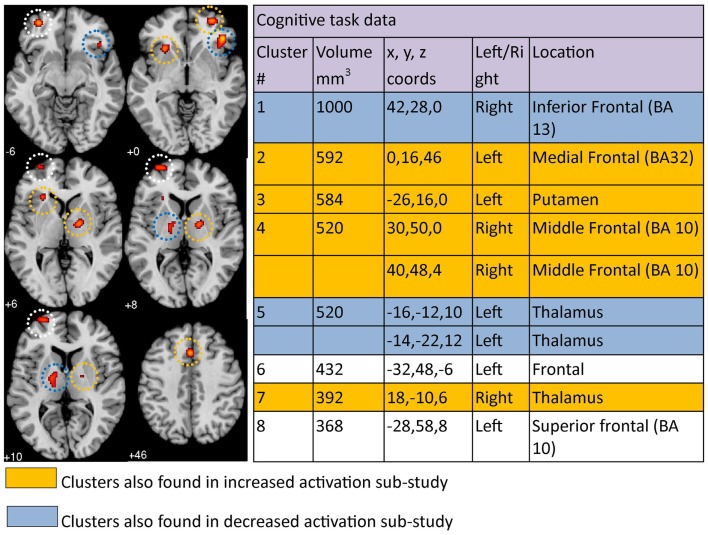
**Regions where depressed patients were altered in activity patterns compared to controls when performing a cognitive processing task**. Clusters listed in the table are taken from the primary meta-analysis comprising all altered co-ordinates. Cluster regions highlighted in orange signify brain areas where depressed patient activation was increased compared to controls. Cluster regions highlighted in blue signify brain areas where patient activation was decreased compared to controls. Co-ordinates (*x*, *y*, *z*) are reported in MNI space. The number under each brain slice displayed is the MNI “*z*” co-ordinate.

**Figure 4:**
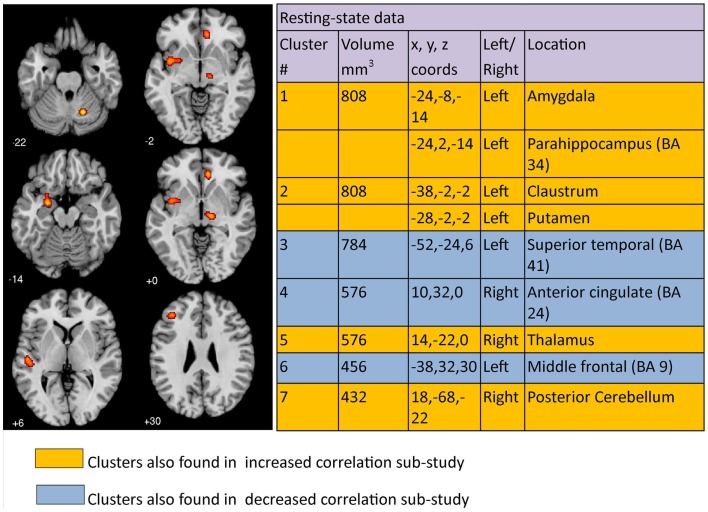
**Regions where depressed patients were altered in correlated brain activity compared to controls during resting condition**. Clusters listed in the table are taken from the primary meta-analysis comprising all altered co-ordinates. Cluster regions highlighted in orange signify brain areas where depressed patient correlated activity was increased compared to controls. Cluster regions highlighted in blue signify brain areas where patient correlated activity was decreased compared to controls. The number under each brain slice displayed is the MNI “*z*” co-ordinate.

In the current study, ALE meta-analysis of the results in each category produced a number of clusters localized to a range of different brain areas. Neither hemisphere appeared to dominate in relation to areas activated (Figures 2–4).

### Emotion task meta-analysis

Using the co-ordinates from 29 emotional task papers (which included a total of thirty studies) and the GingerALE meta-analysis technique, 5 significant clusters of difference between patients with clinical depression and healthy controls were identified across studies. This was after using the 672 mm^3^ threshold recommended by the ALE software (Figure 2). Cluster 1 had three major peaks (or areas of maximum likelihood of difference in depressed patients) that were localized to the left thalamus as well as the left parahippocampal area in BA27 and BA28. Cluster 2 had major peaks in the left amygdala and globus pallidus. Cluster 3 included the right anterior cingulate (BA 25) and the right putamen. The other two clusters were localized to the right amygdala and right insula (BA 13). Clusters are displayed on a template brain (Figure 2).

Results from our exploratory ALE meta-analysis that included studies with co-ordinates that were either *increased or decreased* in depressed patients relative to controls produced similar results, with clusters identified mostly overlapping clusters reported in the primary study (Figure 2). The clusters from the increased/decreased activity meta-analyses are indicated in color in the table sections of Figures 2–4, with orange indicating clusters that were identified in the *increased* activation/correlation meta-analyses and blue indicating clusters identified in the *decreased* activation/correlation meta-analyses. The clusters are also circled with matching colors on the brain images. In the emotion study, clusters in the left thalamic/parahippocampal and left amygdala/globus pallidus regions were also found in the separate meta-analysis of *increased* activation during emotional processing. In addition, clusters identified in the right anterior cingulate/putamen and right amygdala were also found in the meta-analysis of *decreased* activation, while the right insula was not found in either sub-study of increased or decreased activation.

### Cognitive task meta-analysis

The co-ordinates from 15 cognitive task papers (which included a total of 19 studies) comprised the cognition meta-analysis. Eight significant clusters were identified above the recommended 368 mm^3^ threshold (Figure 3). The eight clusters were localized to the right inferior frontal region (BA13), left medial frontal area (BA32), left putamen, right middle frontal area (BA 10), left thalamus, left frontal area, right thalamus, and left superior frontal area (BA 10). Activation was *increased* in the left medial frontal, left putamen, right middle frontal, and right thalamus, but *decreased* in the right inferior frontal and left thalamus, based on our separate cognition meta-analyses of studies reporting increased and decreased activation. Clusters found in the left frontal and left superior frontal areas in our primary meta-analysis were not found in either sub-study of increased or decreased cognitive clusters. Results can be viewed in Figure 3.

### Resting meta-analysis

The co-ordinates of significant differences in correlated brain activity or connectivity between clinically depressed and healthy controls from 21 resting condition papers, each reporting 1 study, when analyzed using GingerALE meta-analysis, yielded 7 clusters >376 mm^3^ (Figure 4). Cluster 1 had two major peaks, one in the left amygdala and one in the left parahippocampal area (BA34), while cluster 2 had peaks in the left claustrum and putamen. The remaining clusters had maximum peak voxels in the left superior temporal region, right anterior cingulate, right thalamus, left middle frontal area, and right cerebellum. *Increased* correlated activity was observed in the left amygdala, left parahippocampus, left claustrum, left putamen, right thalamus, and right posterior cerebellum. In comparison, correlated activity was *decreased* in left superior temporal, right anterior cingulate, and left middle frontal regions under resting conditions. Clusters can be seen mapped to a template brain (Figure 4).

### Analysis of overlap and divergence between clusters across meta-analyses

**Figure 5:**
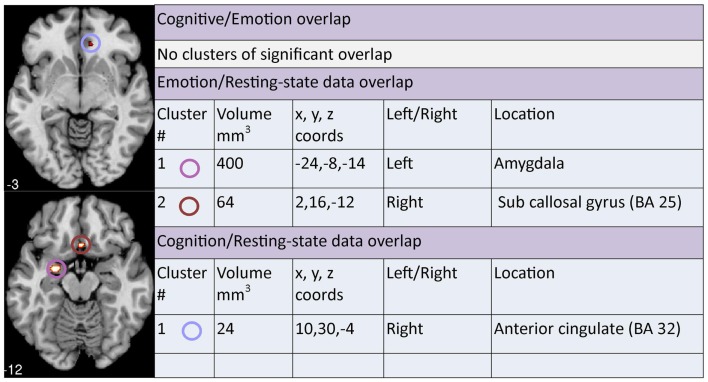
**Areas of significant overlap between emotion, cognition, and/or resting-state meta-analyses**. Co-ordinates (*x*, *y*, *z*) are reported in MNI space. The number under each brain slice displayed is the MNI “*z*” co-ordinate.

**Figure 6:**
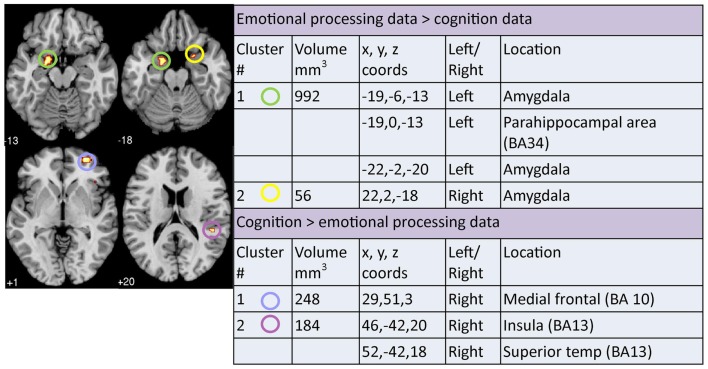
**Areas of significant divergence between areas of altered activation when performing an emotion or cognitive-based task in depressed patients compared to controls**. Co-ordinates (*x*, *y*, *z*) are reported in MNI space. The number under each brain slice displayed is the MNI “*z*” co-ordinate.

A contrast analysis was used to investigate statistically significant overlap and divergence between the clusters generated by the three meta-analyses: emotion, cognition, and resting. There were no areas of significant overlap between *emotion and cognition* meta-analyses (Figure 5). Significant divergence was, however, found in the left amygdala/parahippocampal (BA34) area, close to face processing areas, and right amygdala more in the emotion than the cognition meta-analyses; and in the right medial frontal (BA10) and right superior temporal/insula area (BA13) more in the cognition than the emotion meta-analyses (Figure 6).

**Figure 7:**
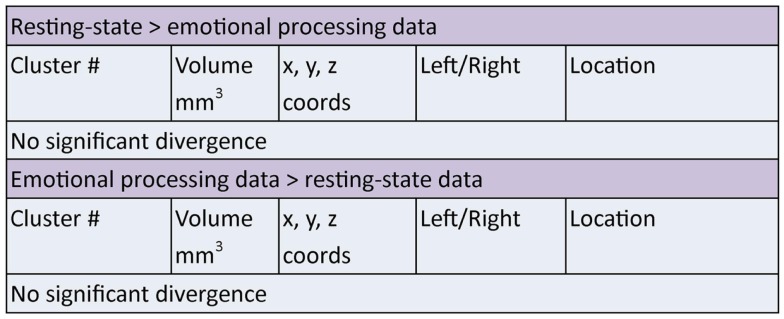
**Areas of significant divergence between areas of altered connectivity during resting-state and when performing an emotion task in depressed patients compared to controls**. Co-ordinates (*x*, *y*, *z*) are in MNI space.

Two clusters of significant overlap were found when comparing the output of the ALE meta-analysis of the *resting and emotion* conditions (Figure 5). These clusters were found in the left amygdala and right sub callosal gyrus (BA 25). There was no significant divergence between the resting and emotion meta-analysis (Figure 7) supporting the interpretation that similar brain regions are altered in depressed patients under resting and emotional processing conditions.

**Figure 8:**
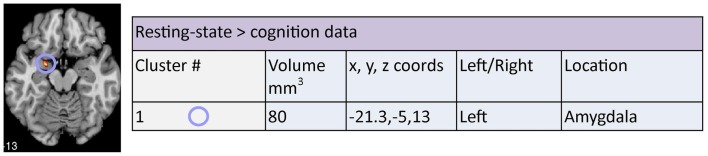
**Areas of significant divergence between areas of altered connectivity during resting-state and when performing a cognition task in depressed patients compared to controls**. Co-ordinates (*x*, *y*, *z*) are reported in MNI space. The number under each brain slice displayed is the MNI “*z*” co-ordinate.

Finally, a cluster of significant overlap was found in the right anterior cingulate (BA 32) when comparing *resting and cognition* meta-analysis cluster outputs (Figure 5). In addition, significant divergence occurred in the left amygdala in resting more than the cognition meta-analyses (Figure 8).

A descriptive exploratory summary of brain areas altered across all three meta-analyses (emotion, cognition, and resting) is shown in Table 2 using common brain area names. As indicated, there were a few areas where two meta-analyses had clusters in the same brain areas but none where all three had clusters in the same brain area when using the recommended threshold. However, after further review of the data for trends, using a reduced cluster size threshold to 100 mm^3^, some clusters were identified in the same brain region across all three tasks. Clusters were present in the right anterior cingulate in all three meta-analyses, although in the cognitive meta-analysis the anterior cingulate clusters (BA 32/BA24) were 328 and 304 mm^3^, i.e., below the significance threshold cluster cut off of 368 mm^3^. Clusters were also present in the left thalamus in all three meta-analyses but this time the cluster in the resting meta-analysis was below threshold. Some common brain regions were significant but in the different hemispheres across conditions, e.g., significant clusters were found in the right thalamus with the resting and cognitive processing tasks but only in the left thalamus for the emotional processing task. Similarly, significant clusters were found in the left putamen with the resting and cognitive processing tasks but only in the right putamen for the emotional processing task.

**Table 2:** **Clusters identified in the same brain region across at least two meta-analyses**.

Emotion	Cognition	Resting
Right anterior cingulate	(Right anterior cingulate)^a^	Right anterior cingulate
Left thalamus	Left thalamus	(Left thalamus)
Left thalamus	Right thalamus	Right thalamus
(Right putamen)	Left putamen	Left putamen
Left amygdala		Left amygdala
Left parahippocampus		Left parahippocampus
Right insula	Right insula	

*^a^Regions in brackets were present within the named meta-analysis at the exploratory cluster level of 100 mm^3^ but below the ALE suggested cluster threshold*.

## Discussion

### Major findings

Brain regions of altered activation in patients with depression compared to healthy controls varies with task demand, according to our quantitative co-ordinate based meta-analysis of common and divergent brain regions identified under emotion and cognition conditions. There were no overlapping brain areas showing altered activation with depression compared to controls in our quantitative comparison of cognition and emotion meta-analyses. However, there were brain areas of significant divergence. Significant divergence of activation, greater in the cognitive meta-analysis, was observed in the right medial frontal, insula, and superior temporal regions than in the cognitively undemanding emotion meta-analysis. Involvement of these regions has come to be expected during tasks that are more demanding of memory and attention manipulations (Pessoa, 2014), consistent with our findings. Review of the differences in cognitive demand across the emotion and cognition tasks included in our meta-analyses would suggest that our finding is not unexpected and that future studies need to consider the relative demands of emotion and cognition task conditions. The cognitive tasks included in our meta-analysis were quite demanding, involving verbal memory encoding/working memory, through word fluency and Tower of London tasks. The cognitive load and brain regions involved in these type of tasks (Rottschy et al., 2012) is different to that involved in tasks such as recognition and matching of faces, pictures, or words (Posamentier and Abdi, 2003). Our findings support this observation and highlight that there are different brain regions altered in depressed patients compared to controls when performing difficult cognitive tasks compared to simple emotional task conditions.

In relation to simple emotionally valenced tasks, significant divergence was observed in comparison to the cognition meta-analysis; involving greater activation of limbic areas, i.e., bilateral amygdala and parahippocampal area. In addition, simple emotion and resting-state meta-analyses only showed overlap (no divergence), involving amygdala and subcallosal cingulate gyrus (BA 25; also referred to as subgenual cingulate). This is an important result as it highlights alteration of limbic and subcortical areas under both non-taxing emotionally valenced and resting conditions. This was the case even though the emotion studies report alterations in activation while resting-state studies report alteration in the correlation of brain activity such as BOLD fluctuations. Involvement of these subcortical regions during simple emotion compared to cognitively demanding tasks, and during resting conditions might be expected based on task demands and previous meta-analyses (Fitzgerald et al., 2008a; Kühn and Gallinat, 2013). It suggests a level of underlying altered brain activity, previously linked with negative rumination and response to emotionally valenced tasks (Holtzheimer and Mayberg, 2011; Mandell et al., 2014), which is present under non-taxing conditions, irrespective of whether there is a simple emotionally valenced task or not.

Use of the ALE contrast analysis to investigate statistically significant overlap and divergence in emotion, cognition, and resting imaging studies comparing clinically depressed patients and healthy controls is novel. Together our findings emphasize the differences in brain regions altered in depressed patients under different task conditions when performing a higher order more demanding cognition processing task compared to when performing a classic emotionally valenced simple cognitive task or under resting conditions. These findings have implication for interpretation of meta-analysis studies that combine both emotional and cognitive processing tasks in depressed patients. Further, without behavioral data indicating level of difficulty to perform the task or amount of effort for the patient, combined task meta-analyses are more difficult to interpret. Our findings also support the value of conducting separate meta-analyses of cognition and emotion tasks, and resting conditions, with use of contrast analyses to determine overlap and divergence.

Finally, in our exploratory, descriptive analysis across all three neuroimaging meta-analyses, we did not find any common areas of overlap across all three conditions at the cluster corrected level. A trend was, however, noted for the right anterior cingulate and left thalamus regions, as well as in thalamus and putamen of either hemisphere. These findings highlight the involvement of subcortical regions that are implicated in most major models and suggest that activation as well as correlated activity/connectivity from these regions is important.

### Characterization of altered brain regions under separate emotion, cognition, and resting conditions

Our *emotion meta-analysis* showed clusters of altered and *increased* activation in left thalamus/parahippocampus and left amydgala/globus pallidus, while right anterior cingulate/putamen, right amygdala showed a relative *decreased* activation. Activation of right insula was also altered but not identified within our separate increased or decreased activation studies. These areas are involved in information processing (thalamus) and recognition of emotional faces or scenes (parahippocampal gyrus), and processing of memory and emotional reactions (amydgala, anterior cingulate). Our meta-analytic findings highlight the involvement of limbic as well as striatal areas, consistent with over activity of limbic areas in the limbic-cortical model (Mayberg, 2003) and with involvement of subcortical structures and parallel cortico-striato-pallaidal-thalamic loops in the corticostriatal model (Bora et al., 2012). Involvement of right anterior cingulate (BA25), putamen, and the left amygdala are consistent with the findings of Fitzgerald et al. (2008a) for emotion tasks, although increased versus decreased activation varied with induction of positive or negative affect and there were additional brain areas reported by Fitzgerald et al. The brain areas identified as significantly altered in emotional processing in our meta-analysis were also reported as altered in depressed patients in a review of emotional face processing (Stuhrmann et al., 2011). These authors reported hyperactivation to negative and hypo-activation to positive stimuli, particularly in the amygdala, parahippocampal gyrus, putamen, insula, and fusiform face area (Stuhrmann et al., 2011). Increased activation of left amydgala and decreased activation of right amydgala may reflect the positive and negative emotional stimuli employed (see Table 1) or hemispheric specialization, based on evidence that stimulation of right amydgala induced negative emotions while stimulation of left amydgala induced either pleasant or unpleasant emotions (Lanteaume et al., 2007). Voxel-based morphometry has suggested that there are volume changes in the right dorsal anterior insula cortex associated with depression, consistent with involvement of this area (Liu et al., 2014).

Our *cognition meta-analysis* confirms previous findings showing that frontal areas of the brain are commonly altered when performing a cognitively demanding task in patients with depression (Fitzgerald et al., 2008b; Diener et al., 2012). Involvement of frontal areas in the cognition meta-analysis is not surprising since many of these areas, such as the superior, inferior, and medial frontal gyri are known to be involved in complex cognitive processing in control subjects (Egner, 2009). The tasks in the cognitive studies included in our meta-analysis typically involved multi-stage cognitive processing, working memory, and verbally oriented tasks consistent with this interpretation. More specifically, activation in middle and medial frontal regions was *increased* in depressed compared to control subjects, while the inferior frontal region was associated with relative *decreased* activation for depressed subjects. Fitzgerald et al. (2008b) suggested increased activation in prefrontal areas was required by depressed subjects in order to perform the cognitive tasks. Increased activation of certain frontal areas compared to controls, e.g., left medial frontal (BA 32) and right middle frontal (BA 10), support hypotheses based on neural efficiency (Haier et al., 1988; Haier, 2003; Bauer et al., 2014). Decreased activation of right inferior frontal region is consistent with reduced cortical inhibition (Aron et al., 2004) as described in the limbic-cortical model (Mayberg, 2003). Left putamen and bilateral thalamus were also involved in our cognition meta-analysis, consistent with the involvement of these regions in limbic-cortical and corticostriatal models of depression (Mayberg, 2003; Bora et al., 2012). These regions were found in both increased (left putamen, right thalamus) and decreased (left thalamus) activation sub-studies. While involvement of cortical areas is more typically highlighted in relation to cognitive tasks, alteration of these subcortical regions, and in particular bilateral thalami, in depressed patients relative to healthy controls is consistent with the role of the thalamus as a critical component of the frontal cortical–basal ganglia–thalamic circuits. These circuits mediate motivation and emotional drive, planning and cognition for the development and expression of goal-directed behaviors (Haber and Calzavara, 2009). Over-activity in left putamen and right thalamus is consistent with over-activity of limbic areas in the limbic-cortical model of Mayberg (2003).

Our *resting meta-analysis* showed clusters of altered and increased activity/connectivity in left amygdala, left claustrum, right thalamus, and right posterior cerebellum. These findings need to be interpreted in the context that all studies included in the meta-analysis conducted whole-brain methods of analysis, such as ICA. They represent the common regions of altered connectivity between depressed patients and healthy controls during resting conditions, i.e., when the participant is not performing an explicit task. Increased correlated activity of amygdala, claustrum, and thalamus are consistent with the findings of Fitzgerald et al. (2008a) for resting conditions. In addition, we also found decreased correlated activity in left middle frontal, left superior temporal, and right anterior cingulate regions, also consistent with Fitzgerald’s meta-analysis. The similarity of the areas observed is interesting given that we added seven fMRI studies and an ASL study to the meta-analysis, whereas the Fitzgerald et al. (2008a) only included PET and SPECT studies. Hamilton et al. (2012), using their own meta-analysis technique, identified bilateral pulvinar nuclei of the thalamus as having increased activity in depressed patients compared to controls. Similar to Hamilton et al. our results also identified right thalamus as being altered (increased) compared to controls; with left thalamus identified when cluster thresholds were less stringent. The brain areas identified in the resting meta-analysis performed by Kühn and Gallinat (2013) differed from our findings, with increased activity in ventral median prefrontal cortex, left ventral striatum, and left thalamus and decreased activity in left postcentral gyrus, left fusiform gyrus, and left insula. The authors highlighted the need to further explore the behavioral correlates of the observed hyper- and hypo-activation. In summary, increased correlated activity of limbic regions and relative decreased activity of middle frontal regions is consistent with the mood regulating cortico-limbic pathway described by Mayberg (1997, 2003). The core role of increased activity of amydgala is consistent with rumination hypotheses (Mandell et al., 2014). There was only minor evidence of differences in correlated activity/connectivity between the clinically depressed patients and healthy controls in the default network.

### Comparison of emotion, cognitive, and resting meta-analyses: overlap and divergence of brain regions

To our knowledge, this is the first study that uses the contrast meta-analysis to directly compare and contrast separate meta-analysis findings from studies of altered brain activity in depressed patients compared to healthy controls when performing an emotional or cognitive task or resting condition. Diener et al. (2012) performed a meta-analysis that *combined* both emotional and cognitive tasks in the one analysis. Combining these conditions was argued on the premise that the emotion and cognition processing centers interact by accessing identical brain regions in broadly overlapping neural networks involving areas such as the amygdala and the prefrontal cortex (Pessoa, 2008, 2014). The expectation was that similar areas of the brain would be affected in depressed patients performing either an emotional or cognitive processing task. Our findings do not support this hypothesis. Rather, they not only highlight a lack of significant overlap of altered areas in the brain during the simple emotional and more difficult cognitive tasks but also a significant divergence of brain regions. Specifically, our contrast analysis revealed differential and enhanced involvement of medial frontal and insula regions with the cognitively demanding condition while the simple emotionally valenced condition showed greater involvement of bilateral amygdala. These regions of divergence are consistent with expectation based on the emotional and cognitive demands of the tasks. For example, a relatively greater involvement of right medial frontal, insula, and superior temporal regions for the cognition meta-analysis, compared to emotion meta-analysis, is consistent with the role of these areas in demanding cognition and task difficulty (Pessoa, 2008, 2014). Interestingly, Diener et al. (2012) reported hypoactive clusters in anterior insula under combined conditions while we found a greater involvement (divergence) under cognition compared to emotion conditions. Greater involvement of bilateral amygdala and parahippocampal area in the emotion meta-analysis is consistent with involvement of limbic areas associated with mood regulation (Fitzgerald et al., 2008a; Stuhrmann et al., 2011). While together the regions are similar to those described by Diener et al. (2012), here we highlight the differences under the different meta-analysis task conditions.

The overlap observed between task and resting-state conditions is of interest. Brain regions identified under non-specific resting conditions are often similar to those reported under related task conditions (Fox et al., 2007), thus overlap is not surprising, despite differences in imaging methods. Overlap, together with a lack of divergent regions, during simple emotionally valenced tasks and resting condition is of particular interest. It suggests that the resting state for those with depression (compared to controls) is closely aligned with brain areas also altered during simple but emotionally valenced tasks (rather than involving the more typical default network). While it is recognized that the type of brain activity altered (activation versus correlated activity) varies across meta-analyses, the overlap of brain regions provides confirming evidence of the role of these regions at a systems-level. It may also suggest a common underlying alteration in brain state. For example, this commonality may be linked with underlying rumination, a symptom of depression, likely present in non-specific task (resting) conditions as well as during simple emotionally valenced tasks: increased amydgala activity is linked with all types of rumination (Mandell et al., 2014). The lack of significant divergence may also be expected because both tasks are simple, i.e., not taxing on attentional reserves. The amygdala and subcallosal cingulate have also been associated with depression in a number of studies (Clement et al., 2011), with the subcallosal cingulate identified as a target for antidepressant therapies (Hamani et al., 2011). These regions are part of the mood regulating cortico-limbic pathway originally reported by Mayberg (1997), and highlight the role of subcortical structures. The core role of the left amygdala in depression is supported not only by its presence in the resting meta-analysis but also its overlap with the emotion task, and its divergence between emotion and cognition meta-analyses.

Overlap of the right anterior cingulate cortex (BA32) under cognition and resting conditions is also of interest. Anterior cingulate is a generic attention area, has a key mediatory role in the limbic-cortical model (Mayberg et al., 1999; Mayberg, 2003; Disner et al., 2011; Etkin et al., 2011) and is associated with reduction in brain volume in patients with MDD irrespective of mood state (Drevets et al., 2008). Increased activation of anterior cingulate in depressed patients suggests that they may need to work harder under cognitive demanding tasks compared to controls and that connectivity to this region is fundamentally altered, as evidenced during the resting condition. A salience network has been proposed, which has been hypothesized to be involved in switching of attention and the control of other networks within the brain (Seeley et al., 2007; Menon and Uddin, 2010). This network has been proposed to be anchored on the anterior cingulate cortex and given that this area has been identified to be altered in depression it would be of some interest to further investigate depression within the context of the salience network.

When performing a comparison of the meta-analyses of brain areas altered in depressed patients across the three conditions (emotion, cognition, and resting) at recommended cluster size thresholds, we found that there were no common areas of the brain that were significantly altered. This was unexpected *a priori* but is consistent with the lack of significant overlap of the emotion and cognition meta-analyses. Right anterior cingulate and left thalamus were, however, identified across all three meta-analyses in our exploratory analysis. Anterior cingulate is a generic attention area, consistent with underlying low level attention demands of tasks and rest. The thalamus has a key role in relay of sensory information for cognition and emotion states and is part of both the limbic-cortical (Mayberg, 2003) and the cortical–striatal (Bora et al., 2012; Graham et al., 2013) models. In the limbic-cortical model, it plays a role in the reduced coordination with other components of the network (Mayberg, 2003; Drevets et al., 2008). It is known to be a source of the low frequency oscillatory potentials associated with arousal and coordination of cortical activity (Steriade et al., 1993; Malekmohammadi et al., 2014). Right thalamus was also identified in two of three conditions (cognition and resting). The putamen was also commonly identified, across hemispheres. Putamen is a key component of the corticostriatal model and is associated with information flow leading to motor planning and learning (Brovelli et al., 2011). Common areas of altered activity across the less demanding emotional valence and resting state were left parahippocampus, likely associated with the verbal face/object demands of the task, and left amygdala. Right insula was observed across emotion and cognition conditions. Interestingly, the regions are primarily subcortical involving limbic and striatal regions.

Support for the three neurobiological models of major depressive disorder, limbic-cortical, corticostriatal, and the default mode network, has been recently reviewed in the context of functional MRI literature involving cross-sectional studies comparing depressed patients and healthy controls and in treatment studies (Graham et al., 2013). In Graham et al.’s review, emotion, cognition, and resting conditions were combined, limiting the ability to identify the contribution of different task conditions to these complementary models. Our findings support differences in brain regions altered in depressed patients compared to healthy controls for the different task conditions. This was particularly evident for more demanding cognition versus emotion tasks as indicated above. While the main regions altered across our separate meta-analyses spanned regions identified in the three models, new insights of the potential contribution under different conditions may be gleaned. A relatively greater activation of limbic areas, i.e., bilateral amygdala and parahippocampal area in simple emotionally valenced tasks compared to cognitively demanding tasks, as well as overlap of amydgala and subcallosal cingulate in emotion and resting conditions, is consistent with the over activity of these regions described in the limbic-cortical model (Mayberg, 2003). In addition, the greater involvement of medial frontal, insula, and superior temporal regions in depressed patients compared to controls under cognitively demanding conditions compared to simple emotional conditions, may reflect the increased need for inhibition by these regions, also consistent with the limbic-cortical model (Mayberg et al., 1999; Mayberg, 2003). Common involvement of anterior cingulate, thalamus, and putamen across the different task conditions further supports the core role of subcortical striatal and limbic regions and the corticostriatal model (Bora et al., 2012), while overlap between resting and task conditions in amydgala, subcallosal cingulate, and anterior cingulate, highlights increased connectivity of these activated regions. We did not find clear support for changes in the default mode network in our resting condition meta-analysis. This is despite recent research targeting alterations in the default network as a possible marker of the disease (Ino et al., 2011; Baojuan et al., 2013; Silbersweig, 2013; Li et al., 2014). Together our findings provide support for the generally held view that depression is a systems-level disorder affecting integrated pathways linking select cortical, subcortical, and limbic sites (Mayberg, 2007). Our findings also provide support for brain network interactions as suggested by Menon (2011) and Pessoa (2014). This type of model would suggest that demand of the task will determine extent of network activation while clinical depression may dampen the efficiency of function and lead to a difference in brain activity between depressed and healthy controls whether doing a simple emotionally valenced task or lying in resting state. Further systematic investigation of alteration of networks is recommended.

### Medication and clinical assessment

The effect that medication may have on the depressed patient’s imaging results is still not clear. We reviewed the studies for the proportion of clinically depressed patients on medication in the named studies. Results revealed that in the emotion studies 48% of the depressed participants were medication free, while in the cognitive studies only 13% of the patients were medication-free and 43% were medication free in the resting-state studies. The exact effect of medication on the brain networks of patients diagnosed with depression remains relatively unknown in these samples and this may be significant as a variety of medications were used, including medications classified as serotonin-specific reuptake inhibitors, e.g., paroxetine, medications classified as serotonin and noradrenaline reuptake inhibitors such as venlafaxine, and drugs classified as tri-cyclic antidepressants such as amitriptyline. Unfortunately, the proportion of studies in each of three categories that involved medication-free depressed patients was quite low and did not allow for separate meta-analysis.

A range of clinical assessments were used to assess and quantify the level of depression in each patient in each study (Table 1). Although all the tests are generally accepted as measures of depression, there has been some suggestion by Kang et al. (2013) that the Beck depression index and Hamilton depression rating scale are more likely to misclassify patients with depression. This may have had implications for the studies involving these assessments.

### Limitations

The task-based meta-analyses undertaken were categorized as “emotion” or “cognition” based on broad categorization of tasks from the original studies, creating potential problems for interpretation of results. The different tasks within a sub-group will influence the results across studies and the co-ordinates entered. For example, combining studies of simple cognitive but varying emotional stimuli, e.g., emotional face and emotional word tasks, may increase the spread of regions activated, reducing the likelihood of significant activation of a particular region across studies. Unfortunately, it was not possible to obtain a more homogenous group of studies with more similar tasks within the emotional or cognition processing groups. Despite this limitation, identification of common regions across studies is likely to be robust and suggestive of changes associated with depression if the tasks are of similar difficulty. While some studies did specify differences in performance on the task between depressed patients and healthy controls, this variation was not able to be interpreted in the context of our meta-analyses. The fact that some but not all depressed patients were medicated may also have an effect on results. Another limitation is the fact that in our resting meta-analysis, co-ordinates were taken from studies using different imaging techniques, correlation of BOLD fluctuations using fMRI, PET/SPECT with a range of labeling mechanisms (listed in Table 1), and ASL perfusion. Although all measure brain activity, typically via blood flow changes, the fact that different methods are used may have affected the sensitivity of the original results and introduced a level of noise.

### Summary and conclusion

Our quantitative co-ordinate based meta-analyses of brain regions altered in depressed patients compared to healthy controls highlight the role of different brain regions when performing simple emotionally valenced tasks compared to cognitively demanding tasks. In contrast, similar brain regions were altered under emotionally valenced and resting conditions. While findings have been previously reported under each of these conditions, our meta-analyses quantified the overlap and divergence under different task conditions. Identification of common and divergent brain regions under different task conditions provides new insight into the relationship between task condition and the current models of depression as well as the core role of different cortical, subcortical and limbic regions when a systems-level approach is taken. Key regions of overlap identified included amydgala and subcallosal cingulate (under emotionally valenced and resting conditions), and anterior cingulate (under cognitively demanding and resting conditions). In comparison, regions of divergence included bilateral amydgala/parahippocampus (emotion > cognition), medial frontal, insula, and superior temporal (cognition > emotion), and amygdala (resting > cognition). The differential involvement of left amygdala in resting and emotional condition studies but not in cognition studies is noteworthy.

In conclusion, these findings support a relative increase of activity in subcortical limbic and striatal regions under emotionally valenced and resting conditions, while during cognitively demanding tasks medial frontal and insula regions are more active. Over activity of subcortical structures is consistent with cortico-limbic and striatal models. The relative increase of medial frontal and insula regions under cognitively demanding conditions supports the interpretation that depressed patients may need to work harder on the basis of capacity limited performance (Fitzgerald et al., 2008b; Pessoa, 2014). Identification of common and divergent brain regions may better inform current neurobiological models of depression and the mechanisms underlying.

## Conflict of Interest Statement

The authors declare that the research was conducted in the absence of any commercial or financial relationships that could be construed as a potential conflict of interest.
